# Identifying transcriptomic signatures that mediate the causal effect of genotype on Alzheimer's disease

**DOI:** 10.3389/fnins.2026.1716828

**Published:** 2026-03-11

**Authors:** Simran Kaur, Anna L. Tyler, Giovanna L. Durante, Gregory A. Cary, Gregory W. Carter, J. Matthew Mahoney

**Affiliations:** 1 The Jackson Laboratory, Bar Harbor, ME, United States; 2 Department of Biomedical Engineering, Boston University, Boston, MA, United States; 3 Program in Quantitative Biomedical Sciences, Dartmouth College Geisel School of Medicine, Hanover, NH, United States

**Keywords:** Alzheimer's disease, biodomains, causal inference, drug repositioning, ROSMAP, transcriptome

## Abstract

The combined effects of thousands of genetic polymorphisms account for Alzheimer's disease (AD) genetic risk. Most AD polymorphisms affect gene expression. Thus, the transcriptome, the set of all gene expression levels for every gene in the genome, is a major mediator between the genotype to phenotype. This study uses genotypes, transcriptomes, and clinical phenotypes to identify the transcriptomic signature that mediates the causal effect of genotype on AD. By utilizing a causal inference method known as high dimensional mediation analysis (HDMA) on the Religious Orders Study/Memory and Aging Project (ROSMAP) longitudinal cohort, the genotype, transcriptome, and phenotype data were reduced to single scores encoding genotype, transcriptome, and phenotype correlations, and produce a ranked gene list based on putative causal importance of each gene for AD. Analysis of the up- and down-regulated genes prevalent in AD through Gene Ontology (GO) and KEGG databases reveals findings such as up-regulated functions which include angiogenesis and immune responses while down-regulated functions of genes include synaptic activity. Furthermore, utilizing Clue.io to identify candidate drugs to suppress AD-pathology reveals a plausible list of therapeutic candidates, including targeted genes and compounds such as *SMAD3, TM7SF2*, and *ABCB1*, which counteract the transcriptomic signature identified and may block the devastating effects of AD related to inflammatory responses, Aβ-induced toxicity, and neuronal death.

## 1 Introduction

Alzheimer's disease (AD) is a chronic neurodegenerative disease that affects cognitive function and memory. Over 55 million individuals worldwide are affected by dementia, with AD accounting for 60–70% of these cases (World Health Organization: WHO, 2023). Genetically, less than 5% of AD cases are due to single mutations that cause AD by themselves (Tanzi, 2012), with the remainder of cases caused by interactions among many genes and the environment. Over 70 loci with risk variants have been identified (Bellenguez et al., 2022). While 24–33% of AD risk can be attributed to common single nucleotide polymorphisms (SNPs), only 2–6% is accounted for by currently identified SNPs, which has been termed the *missing heritability problem* (Cuyvers and Sleegers, 2016; Sadee et al., 2014). It is now expected that the remaining genetic risk is due to the combined effect of potentially thousands of additional SNPs, each of which contribute small genetic effects (Boyle et al., 2017). In a therapeutic context, it is reported that drug mechanisms with a genetic basis are 2.6 times more likely to be successful than without (Minikel et al., 2024). However, the genetic complexity of AD implicates multiple biological pathways, making target identification and prioritization a significant challenge.

The pathology of AD is multifactorial and includes neuronal death, inflammatory responses, oxidative stress, lipid metabolism, insulin resistance, among others, and these processes are interrelated (Du et al., 2018; Chew et al., 2020; Amin et al., 2022). Correspondingly, we expect the heritable variation in gene expression driving AD to manifest as a transcriptome-wide signature encompassing the correlated biological processes underlying disease. Given that most genetic risk factors for common disease are non-coding, gene expression is expected to be a major mediator in the causal chain of genetic influences leading to AD. Because of the convergence of many genetic effects on transcription, identifying the transcriptomic signatures that mediate the complex genetic background effects on AD clinical phenotypes is a critical task for the mechanistic dissection of AD pathology and the development of novel treatment strategies.

To identify transcriptomic signatures mediating AD severity, we employed the recently developed *high dimensional mediation analysis* (HDMA), which is a dimensionality reduction technique to project, in our case, genomic, transcriptomic, and clinical data into one-dimensional scores and corresponding high-dimensional signatures (Tyler et al., 2025). From these signatures, we used enrichment analyses to identify the key biological processes defining these signatures and performed *in silico* drug repositioning analyses to identify candidate drugs to potentially reverse these signatures.

## 2 Materials and methods

### 2.1 ROSMAP data acquisition and preprocessing

**Figure 1:**
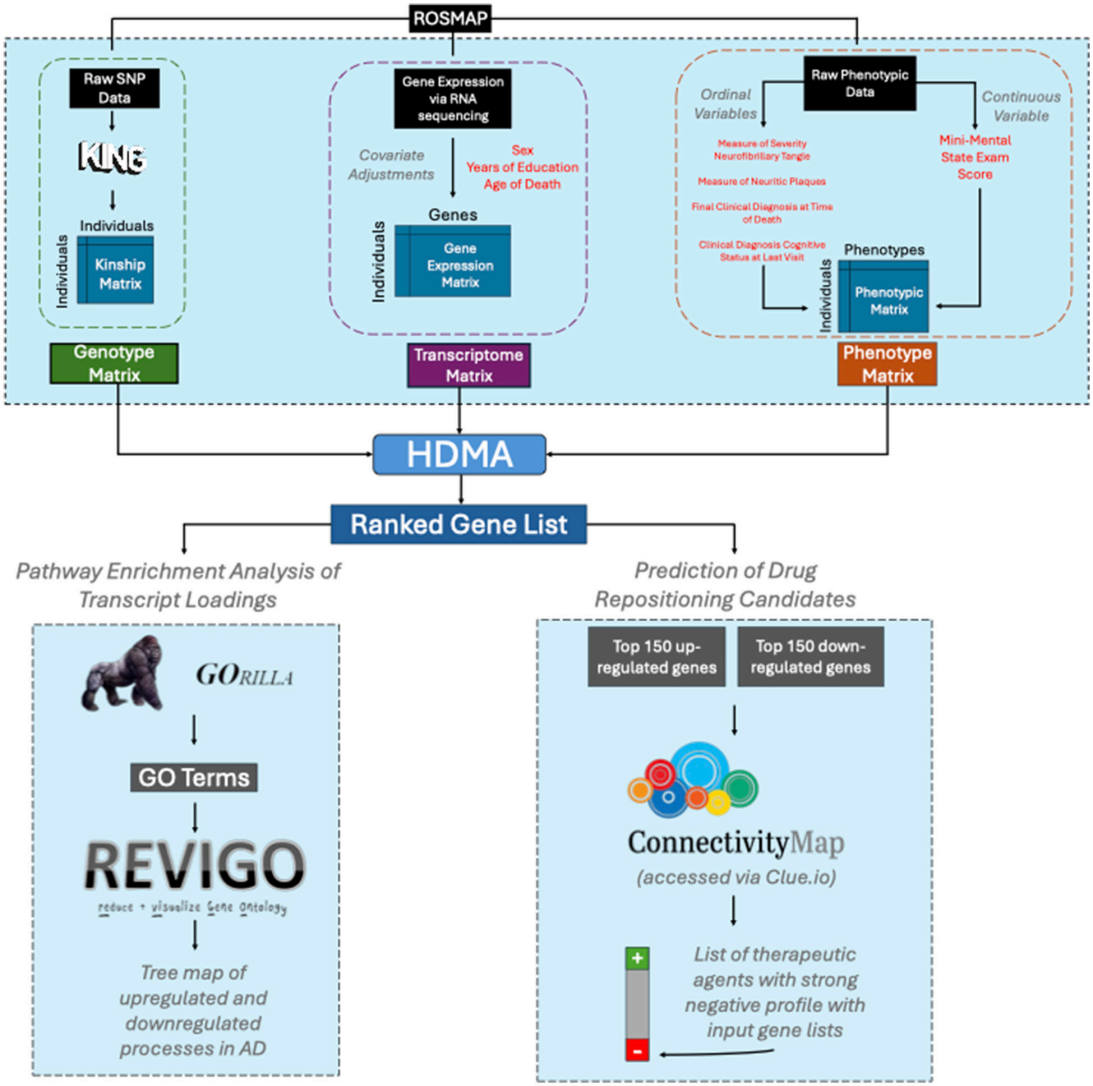
Pipeline of processed genotype, transcriptome, phenotypes inputted into HDMA. A matched 477 individuals from the ROSMAP cohort comprising of genotype, transcriptome, and phenotype matrices were inputted into HDMA to produce a ranked gene list where downstream processes consist of pathway enrichment analyses (GOrilla, Revigo) and prediction of drug repositioning candidates (Clue.io).

As shown in the pipeline in Figure 1, the genotype, transcriptome, and clinical phenotype matrices were obtained from the ROSMAP study from the AMP-AD Knowledge Portal [Sage Bionetworks (AMP-AD Knowledge Portal), no date]. Genotype data were derived from SNP genotyping arrays. Transcriptomic profiles were from the dorsolateral prefrontal cortex of the brain post-mortem (De Jager et al., 2018). The combined covariate and phenotype dataset are available on Synapse as syn3191087 (Greenwood et al., 2020). The genotype matrix and gene expression matrix are available on Synapse as syn3157325 and syn23650893 respectively. The ROSMAP samples analyzed in this study were derived from the same ROSMAP cohort as described by Milind et al. (2020). Additional summaries of demographic information such as sex, diagnosis, and RNA-seq samples are provided in Supplementary Tables 1, 2 of that publication.

The raw genotype matrix was used to compute a kinship matrix, which encodes genetic similarities amongst the cohort using the KING relationship inference software Manichaikul et al., (2010). The symmetric, square kinship matrix was used for all genetic analyses downstream. To generate gene expression matrices, we logarithmically transformed the normalized read counts (available on Synapse as syn8691134) from RNA sequencing and adjusted the gene expression levels using sex, years of education, and age at death as covariates. Finally, we scaled each transcript to have a mean of zero and a standard deviation of one. The purpose of including covariates was to control for non-genetic factors driving variation in AD phenotypes to focus on the underlying genetics of the individuals in the study.

The raw clinical data included the mini-mental state exam score at last visit (MMSE; coded as “cts_mmse30_lv” in the ROSMAP dataset), the Braak score of severity neurofibrillary tangles (Braak; coded as “braaksc” in the ROSMAP dataset), the CERAD score of neuritic plaques (CERAD; coded as “ceradsc” in the ROSMAP dataset), the clinical diagnosis cognitive status at last visit (Cog LV; coded as “dcfdx_lv” in the ROSMAP dataset), and the final clinical diagnosis at time of death (Cog death; coded as “cogdx” in the ROSMAP dataset).

Aside from the mini-mental state exam, the phenotypic variables are ordinal variables where the order among values but not the numeric value matters. To account for this, we coded the ordinal variables in the phenotype matrix using binary dummy variables for each level of the ordinal scale, filling in 1s up to the assigned scores for the individual. For example, for Cog death, which had six levels, an individual who received a 4 for this score is encoded as a binary vector with 1s in the first four components and the zeros in the fifth and sixth components to show the individual meets the criteria for all scores up to four. This ordinal encoding preserves the order structure but does not assign equal weight to the differences between levels.

### 2.2 High-dimensional mediation analysis

To reduce the dimensions of genotype, transcriptome, and phenotype matrices, we used a recently developed tool called high-dimensional mediation analysis (HDMA; Tyler et al., 2025). The purpose of HDMA is to simultaneously correlate genotype to transcriptome and transcriptome to phenotype to identify the biological processes that mediate the effect of genotype on phenotype. Briefly, the inputs to HDMA are the genotype, transcriptome, and phenotype data matrices and the output is a set of *scores* and *loadings* for each data matrix. The genome, transcriptome, and phenome scores encode the values for each individual of the projection of the high-dimensional data set to a single dimension. The scores are computed so that their correlations are maximally consistent with the perfect mediation model. Biologically, this ensures that the transcriptomic score is simultaneously correlated with both genome to phenome scores and, further, that the phenome score is uncorrelated with the genome after controlling for the effect of the transcriptome score. This latter condition is the definition of a *perfect mediator* (Bollen, 1989; Shipley, 2000).

Formally, HDMA constructs scores—*g, t*, and *p*—as linear combinations of the measured features


$$\begin{array}{l} g=K_{G}l_{G}\\ t=\;K_{T}l_{T}\\ p=\;K_{P}l_{P} \end{array}$$

where *K*_*G*_ is the kinship matrix, $K_{T}=XX^{t}$ is the transcriptome Gram matrix (*X* is the individual by gene matrix), and $K_{P}=PP^{t}$ is the phenome Gram matrix (*P* is the individual by phenotype matrix), and *l*_*G*_, *l*_*T*_, and *l*_*P*_ are *loading vectors* defining the weighted combinations of raw data features to generate univariate scores. Because the total scale for *g, t*, and *p* is arbitrary, we scale the loading vectors to ensure that the variance of all scores is one. In HDMA, the loading vectors are learned from data by the criterion that they maximize the likelihood of perfect mediation model, which is defined by the following structural equations


$$\begin{array}{l} t=\alpha g+\;\epsilon_{t}\;\\ p=\;\beta t+\epsilon_{p}\; \end{array}$$

where α and β are unknown *structural parameters* and ϵ_*t*_ and ϵ_*p*_ are assumed normally distributed with mean zero. The structural equations above define a multivariate Gaussian model with the log-likelihood given by


$$\begin{array}{l} L=tr\left(S\Sigma^{-1}\right)+log\left(det\left(\Sigma \right)\right) \end{array}$$

where *S* represents the covariance matrix among the scores, represents the model-implied covariance matrix for the perfect mediation model, and tr and det denote the trace and determinant of a matrix, respectively. We optimize the loadings and structural parameters by minimizing the negative log-likelihood. As we have shown in Tyler, et al., this optimization can be readily performed using an algorithm that iteratively updates between optimizing the structural parameters and the loadings. The loading optimization problem is equivalent to a multivariate analysis problem called generalized canonical correlation analysis, which we solved using the R package “RGCCA” [Comprehensive R Archive Network (CRAN), 2023]. Complete mathematical and algorithmic details, including an R implementation, are available in Tyler, et al. which we used in this study.

The input of HDMA accepts matrices that have the same number and order of individuals. A total of 477 individuals were common among the kinship, gene expression, and phenotype matrices and included no missing or NA values. The three matrices were input into HDMA and the genotype, transcriptome, and phenotype scores were computed based on the optimal loadings.

### 2.3 Pathway enrichment analysis of transcript loadings

Using the loadings to each gene in the transcriptome, we ranked genes based on their causal importance to AD. We converted the ensembl gene names to their appropriate common symbols using biomaRt (Durinck et al., 2009) and did not analyze ensembl gene ids with a missing name. The ranked gene list was used to create an ascending, down-regulated, and descending, up-regulated, list of genes based on their transcript loadings. We used GOrilla to identify biological pathways associated with these ranked gene lists (Eden et al., 2009). The entire ranked gene list from HDMA, comprising 14,710 genes (Supplementary Table 2), was input into GOrilla in separate ascending and descending lists reflecting the down-regulated and up-regulated genes respectively. The output included biological terms relevant to AD in the form of gene ontology (GO) terms. To condense these GO terms to a manageable number of up and down-regulated biological processes, we inputted the GOrilla output into Revigo, which groups similar terms together (Supek et al., 2011). The Revigo output is a tree map, i.e., a visual display of clusters of enriched GO terms, from which we can read off specific processes (Supek et al., 2011).

In parallel, we used the lists of genes ordered by causal importance to AD for gene set enrichment analysis (GSEA) using the gseGO function from the clusterProfiler R package (v4.12.6; Xu et al., 2024) against the org.Hs.eg.db annotation database (v3.19.1; doi: 10.18129/B9.bioc.org.Hs.eg.db). The ascending (down-regulated) and descending (up-regulated) lists of ensemble gene IDs were given arbitrary ranks based on the order in each list and the order was used as a ranking statistic for GSEA in positive score mode. Significantly enriched GO terms were mapped to the TREAT-AD biological domains (syn25428992.v10; Cary et al., 2024), which organize GO terms into common AD associated molecular endophenotypes.

### 2.4 Prediction of drug repositioning candidates

To identify candidate drugs to suppress AD pathology, we used Clue.io (Subramanian et al., 2017) to compare cellular signatures of responses to therapeutic agents to the top 150 up- and down-regulated genes. Briefly, Clue.io searches for therapeutic response signatures that down-regulate the gene that are up in disease, and vice versa. The Clue.io database includes signatures from various cell types, multiple types of candidate therapies (e.g., small molecules or short hairpin RNAs), and a range of dosages. The output of Clue.io included a list of candidate therapeutic agents that had a significant anti-correlation with the input gene lists.

## 3 Results

To identify a transcriptomic signature of the biological processes that mediate clinical disease severity in AD, we applied HDMA to data from the Religious Orders Study/Memory and Aging Project (ROSMAP). ROSMAP is a longitudinal cohort study of dementia and other chronic diseases of aging (Bennett et al., 2018; De Jager et al., 2018). Participants in the study voluntarily agree to medical and psychological evaluations every year and brain donation postmortem, allowing histopathological examination and bulk transcriptomics data to be generated from the dorsolateral prefrontal cortex. ROSMAP allows for an individual-by-individual view of an aging population with varying cognitive status, which includes no cognitive impairment (NCI), mild cognitive impairment (MCI), and AD.

The input of HDMA requires three matrices—genotype, transcriptome, clinical phenotypes—from the same set of individuals. The purpose of HDMA is to simultaneously correlate genotype to transcriptome and transcriptome to phenotype to identify the biological processes that mediate the effect of genotype on phenotype. From this putative causal flow, transcriptomic signatures of AD can be generated and genes ranked based on casual importance to AD. We analyzed the ranked gene list to identify the mediating biological processes driving AD as well as to nominate candidate therapies and drug targets perturbations with the potential to reverse these transcriptomic changes (Kanehisa et al., 2016; Subramanian et al., 2017).

### 3.1 HDMA identifies a heritable transcriptomic signature associated to AD clinical phenotypes

**Figure 2:**
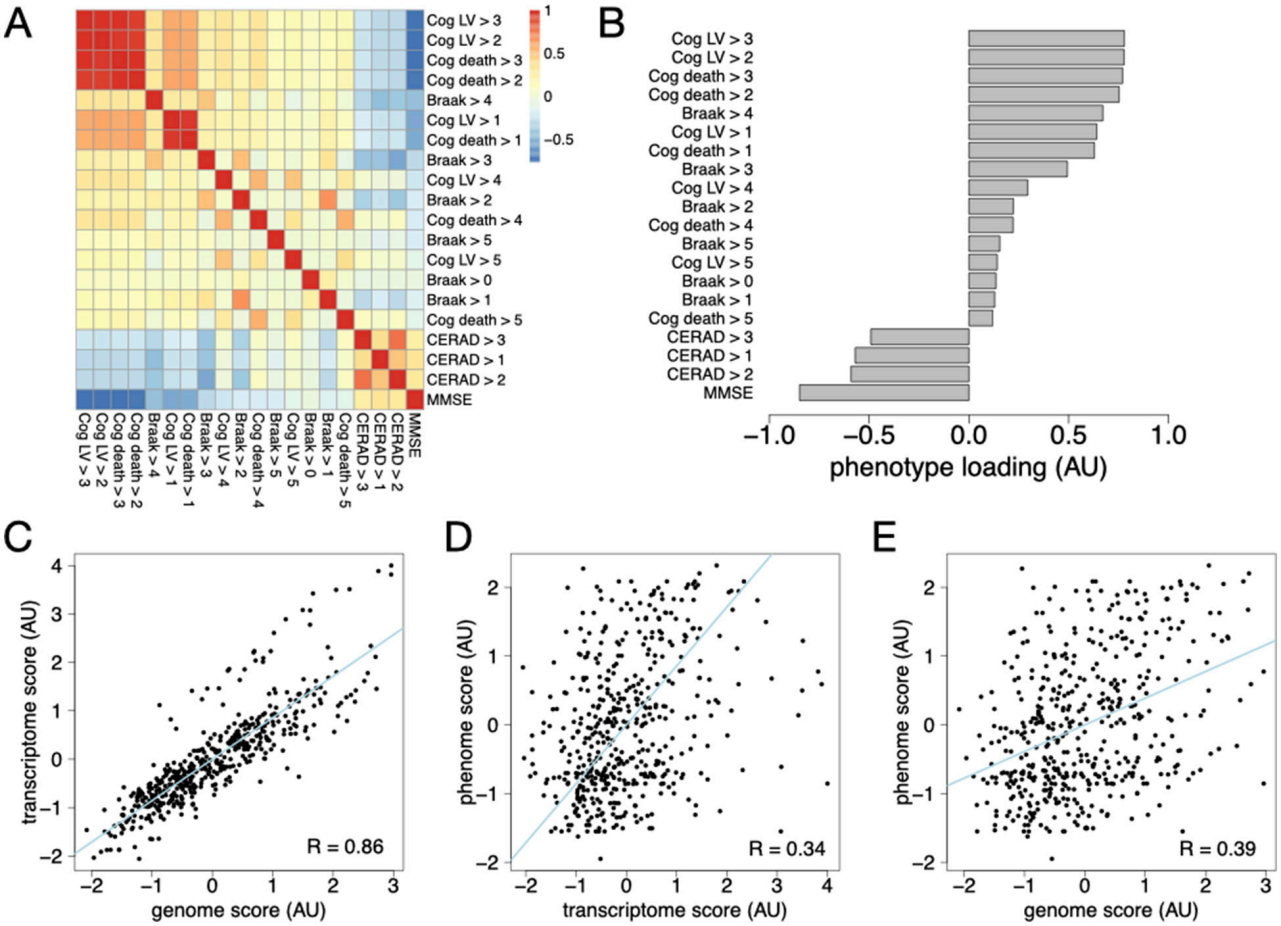
HDMA analysis of the ROSMAP cohort. **(A)** The correlation structure among clinical phenotypes reflected known covariation among cognitive decline and amyloid and tau pathology. **(B)** The phenotype loadings defining the composite phenome score reflect the correlated variation in cognitive scores and amyloid and tau pathology, yielding an overall AD severity score. **(C)** The HDMA-derived genome and transcriptome scores were highly correlated (*R* = 0.85, *p* < 2.2^−16^). **(D)** The HDMA-derive transcriptome and phenome scores were modestly but significantly correlated (*R* = 0.34, *p* = 3.3 × 10^−14^). **(E)** The HDMA-derive genome and phenome scores were modestly but significantly correlated (*R* = 0.38, *p* < 2.2^−16^).

A subset of 477 individuals from the ROSMAP cohort had matched genotypes, transcriptomes, and clinical phenotypes. The clinical phenotypes were highly correlated, as expected (Figure 2A). For example, having a cognitive decline score at last visit > 3 (Cog LV > 3) was correlated with having a cognitive decline score at last visit > 2 (Cog LV > 2), as well as having a cognitive decline score at death > 3 (Cog death > 2). The first two principal components of the trait matrix explain 48% of the total variance across all traits (Supplementary Figure 1), demonstrating a high degree of covariation across all traits. We applied HDMA to this data set to generate composite scores for each data set. The phenome score is a composite trait built from a linear combination of the clinical traits, where the phenotype loadings define how much each trait contributes to the composite score (Figure 2B). The most positive loadings were on Cog LV > 3, Cog LV > 2, Cog death > 3, Cog death > 2, and Braak > 4. The cognitive scores were assessed through cognitive exams and consensus among multiple healthcare providers. The Braak score, in contrast, requires post-mortem examination of brain tissue for tau pathology in neurons. Braak > 4 was modestly correlated with each of the high-loading cognitive scores (Figure 2A), demonstrating that the Braak score and cognitive outcomes are separately contributing to the overall phenome score. The most negative loadings were on MMSE and CERAD scores. High scores on MMSE correspond to higher cognitive performance, while high scores on CERAD correspond to lower amyloid pathology burden in post-mortem examination. These scores are negatively correlated with the cognitive decline scores with positive loadings. Thus, the phenome score is an overall severity score that integrates measures of late-life cognitive function with post-mortem pathological assessment.

The genome and transcriptome scores were strongly correlated (*R* = 0.85, *p* < 2.2^−16^; Figure 2C), demonstrating that the mediating transcriptomic signature was highly heritable. The transcriptome and phenome scores were modestly but significantly correlated (*R* = 0.34, *p* = 3.3 × 10^−14^; Figure 2D). This correlation suggests that the transcriptome score represents biological processes driving clinical variation in AD. From this we conclude that the transcriptome score reflects mediating processes driving heritable variation in AD severity.

The genome and phenome scores were also modestly but significantly correlated (*R* = 0.38, *p* < 2.2^−16^; Figure 2E), which is larger than expected under the perfect mediation model, i.e., *R*_exp_= 0.85 × 0.34 = 0.29. This is likely due to direct effects of the genome on AD severity that are mediated through other mechanisms than those represented by late-stage disease in the dorsolateral prefrontal cortex. Nevertheless, the transcriptome score reflects the latent variable defined by the genome score and can be used to mechanistically interpret the effect of genetic risk.

### 3.2 Enrichment analysis of the mediating transcriptomic signature is enriched for AD-related pathways

As with the phenome score, the loadings on individual genes in the transcriptome score determine their individual influence on the overall composite score. High expression of genes with positive (negative) loadings, by definition, contribute to increasing (decreasing) the transcriptome score, which itself is positively correlated with the phenome score. Thus, we infer that the sign of the loading defines the direction of putative causal effect on the aggregate phenome score.

**Figure 3:**
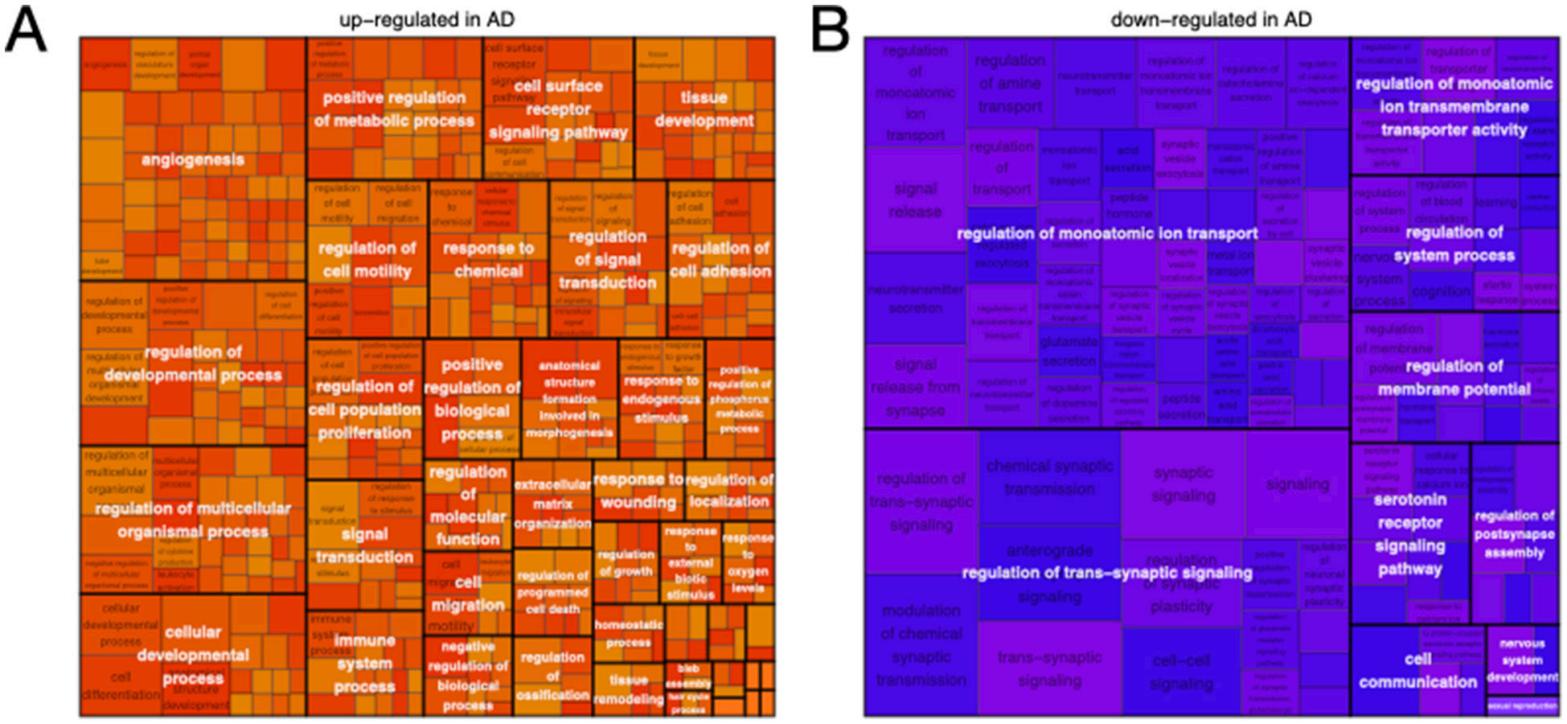
Enrichment analysis of up- and down-regulated genes defining the HAD-derived transcriptomic score. **(A)** A tree map of the up-regulated genes shows enrichment for biological processes including angiogenesis, immune processes, tissue development and remodeling, extracellular matrix organization and response to wounding, regulation of programmed cell death, and response to oxygen levels. **(B)** A tree map of the down-regulated genes shows enrichment for biological processes involved in synaptic function and membrane excitability.

To gain a system-level understanding of the transcriptomic score, we performed pathway enrichment analysis using GOrilla and summarized the enrichment results using Revigo (Eden et al., 2009; Supek et al., 2011). The up-regulated pathways in AD show strong enrichment for biological processes including angiogenesis, immune processes, tissue development and remodeling, extracellular matrix organization and response to wounding, regulation of programmed cell death, and response to oxygen levels (Figure 3A). Each of these processes is known to be dysregulated in AD, which is associated with neural inflammation abnormal angiogenesis, neuronal death, abnormal extracellular matrix, brain hypoxia (Cary et al., 2024). In contrast, the down-regulated genes are enriched for processes involved in synaptic function and membrane excitability, reflecting the higher level of healthy neural activity in the brains of subjects without AD (Figure 3B).

**Figure 4:**
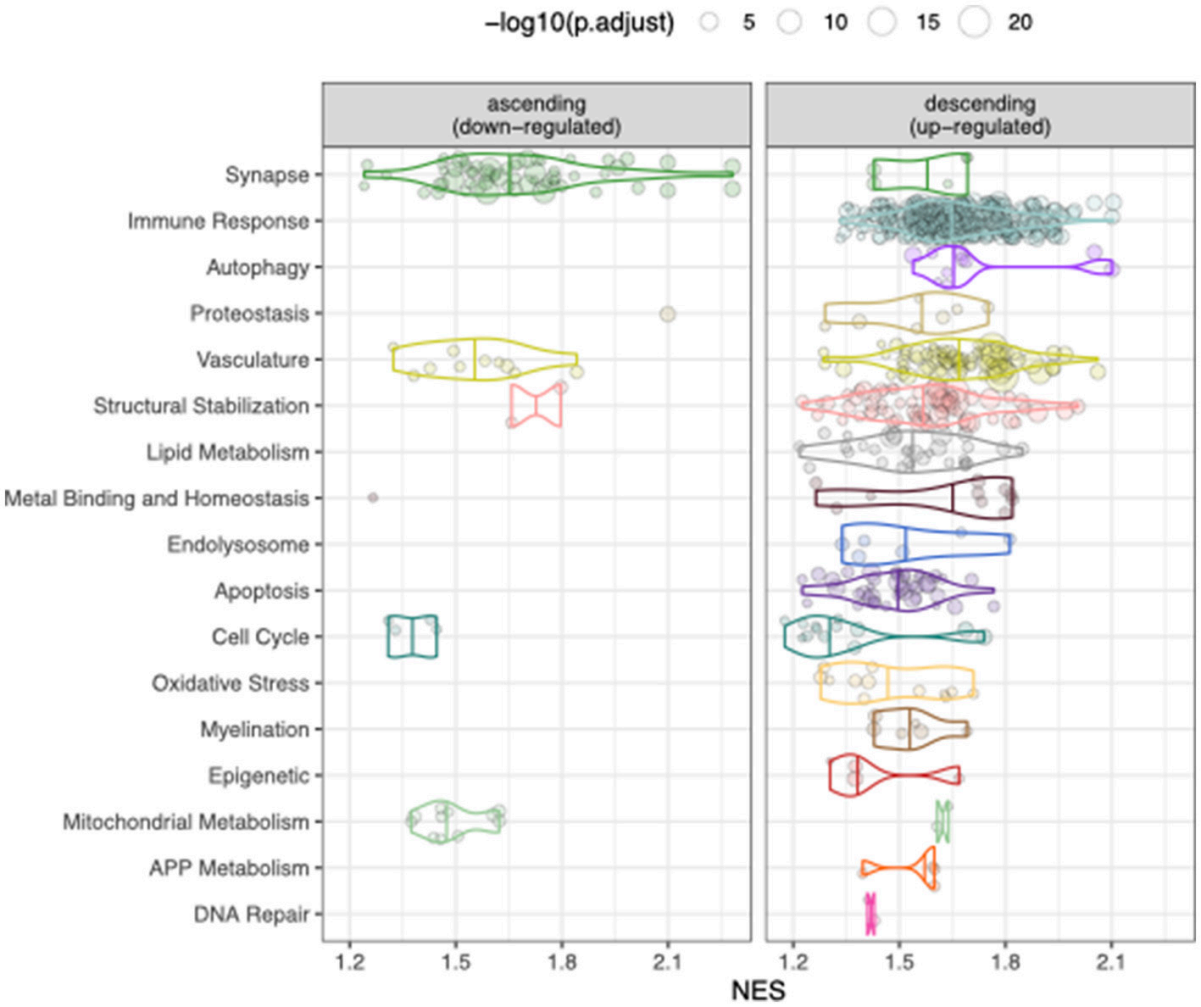
Biological domains enriched using HAD-derived transcriptomic score. GSEA results showing significantly enriched GO terms (points) from each biological domain (*y*-axis). The normalized enrichment score (NES, *x*-axis) reflects the degree to which genes from each term are concentrated at the top of the ranked list. Point size corresponds to the –log10 adjusted *p*-value for enrichment.

Genes involved in the above processes have been bioinformatically curated into the AD Biodomains (Cary et al., 2024). To further interpret our transcriptomic signature, we performed gene set enrichment analysis and mapped the enriched GO terms onto the biodomains (Figure 4, Supplementary Table 1). The strongest enrichments among the up-regulated transcriptomic mediators are from the Immune Response, Autophagy, Vasculature, and Structural Stabilization biodomains, among others, and include “regulation of phagocytosis, engulfment” (NES 2.1, adjusted *p*-value 4.1 × 10^−4^), “coronary vasculature morphogenesis” (NES 2.1, adjusted *p*-value 3.8 × 10^−4^), and “cell-cell adhesion mediated by integrin” (NES 2.0, adjusted *p*-value 1.2 × 10^−3^). The strongest enrichments among down-regulated transcriptomic mediators are from the Synapse biodomain and include “regulation of synaptic vesicle fusion to presynaptic active zone membrane” (NES 2.3, adjusted *p*-value 1.6 × 10^−4^), “regulation of neurotransmitter receptor activity” (NES 2.0, adjusted *p*-value 2.2 × 10^−4^), and “postsynaptic actin cytoskeleton organization” (NES 1.9, adjusted *p*-value 2.7 × 10^−2^). Collectively, the strongest putative transcriptomic mediators of genetic effects on AD phenotypes map to up-regulation in processes related to phagocytosis, angiogenesis, and cell adhesion and to down-regulation in processes related to the synaptic vesicle cycle (Supplementary Figures 2, 3). Interestingly, genes that have been associated with LOAD using GWAS and expression quantitative trait locus (eQTL) co-localization analysis were not significantly enriched in the loadings, suggesting that HDMA identified a complementary signature to that identified by standard gene prioritization techniques (Supplementary Figure 4).

### 3.3 *In silico* drug repositioning analysis implicates small molecules and gene perturbations to reverse the putative mediation signature

The transcriptomic signature from HDMA represents multiple correlated biological processes that putatively drive variation in AD severity. While it is heritable, this signature is also highly polygenic, reflecting the polygenicity of AD itself. To identify potential therapeutic approaches to suppress this signature, we used the transcript loadings as a query to the clue.io database (Subramanian et al., 2017). Briefly, the cluie.io database uses the ConnectivityMap approach (Subramanian et al., 2017) to match a query signature (i.e., a ranked gene list) to corresponding gene expression response profiles in human cell lines treated with drug compounds or gene-targeting treatments (collectively called perturbagens). Perturbagens with highly anti-correlated response signatures to the query are prioritized as potential therapeutic strategies to treat AD.

## 4 Discussion

**Figure 5:**
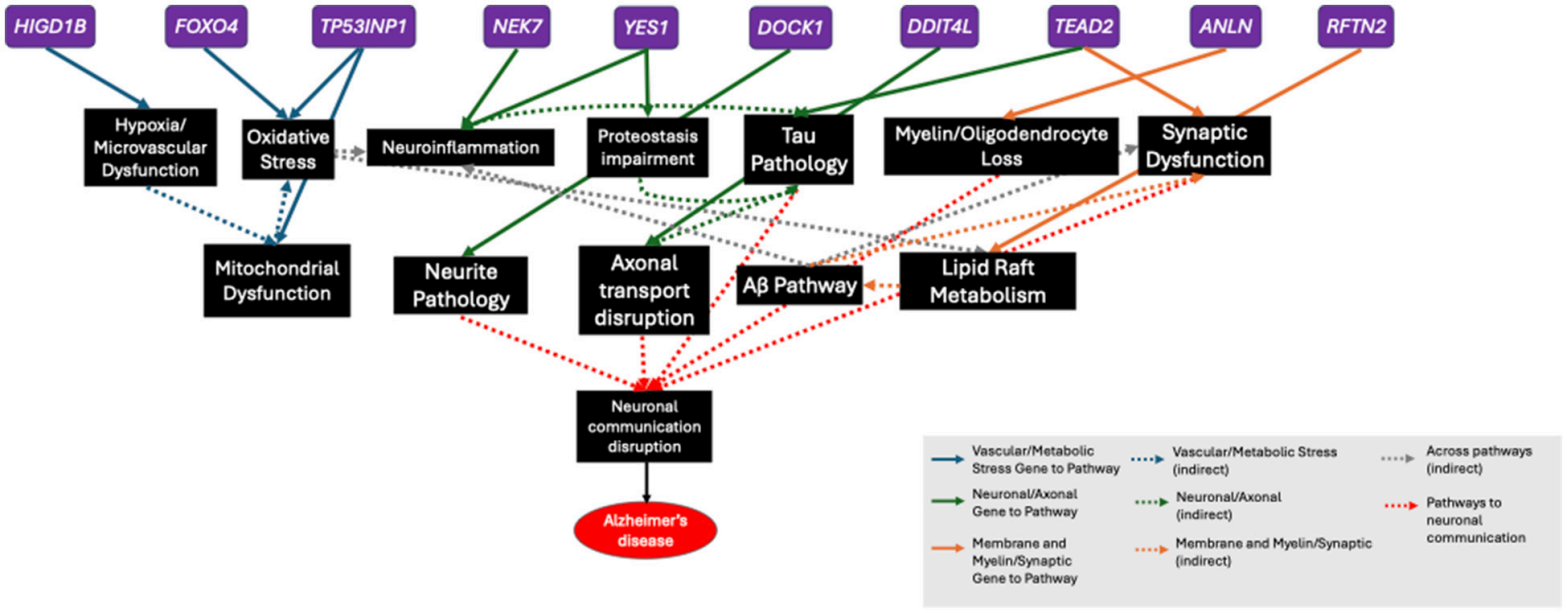
Inferred direct and indirect causal associations among top ten up-regulated genes from HDMA and biological pathways to Alzheimer's disease. The top ten up-regulated genes from HDMA (purple) are linked to a diverse range of biological pathways (black) mentioned in Section 4.1 through direct and indirect gene to pathway mediators to Alzheimer's disease.

There exists speculation on the causal role of several pathways associated with AD (Calabrò et al., 2020). In this study, we used HDMA to identify a transcriptomic signature that captures the putative causal flow of effects from genome through transcriptome to the AD phenome. Overall, we found a highly heritable transcriptomic signature that is correlated with AD severity, is highly enriched for AD-specific biology, and is predicted to be targetable by existing compounds or gene-targeted therapies. The down-regulated genes in our analysis play key roles in neurotransmitter and synapse activity. Reduced expression of these genes may reflect diminished synaptic function and activity, loss of synaptic structures, and a reduction in neuronal populations. Because bulk RNA-seq does not allow distinguishment between changes in cell-type abundance and altered gene expression within surviving cells, these enrichment domains likely represent a combination of synaptic dysfunction and neurodegeneration. High ranking down-regulated genes such as *CPLX1* and *DOC2A* have been identified through protein quantitative trait locus (pQTL) analysis as contributors to neurologic and psychiatric conditions (Wingo et al., 2025). Indeed, a pQTL associated with decreased abundance of DOC2A protein is implicated as causally connected to AD. Conversely, the up-regulated genes in our analysis implicate oxidative stress, microvascular abnormalities, lipid metabolism, and neuroinflammation as major drivers of AD pathogenesis. Given that the HDMA loading corresponds to the weight of a gene in the transcriptomic score, below we discuss the top ten genes with the highest loadings under the assumption that these are important genes in mechanistically important pathways in AD. Indeed, several top up-regulated genes have been experimentally validated for mechanistic association to AD-related biology (Figure 5, Table 1). Similarly, the top ten down-regulated genes from HDMA have been mechanistically linked to several biological pathways and relationships with AD (Table 2). We stress, however, that the discussion below is only expected to point to pathways in a systemic picture, not that these genes are singularly important relative to high-ranking genes outside the top ten.

**Table 1:** Summary of top ten up-regulated genes from HDMA.

**Gene**	**Function/role**	**Hypothesized contribution to Alzheimer's disease**	**Primary affected pathway**	**Biodomain annotations**	**References**
*FOXO4*	Forkhead box O transcription factor involved in insulin signaling and oxidative stress responses	Dysregulation impairs insulin signaling and glucose metabolism in brain microvasculature, contributing to oxidative stress	Oxidative stress, insulin signaling	Cell cycle, epigenetic, vasculature, oxidative stress	Storz, 2010; Santos et al., 2023; Manolopoulos et al., 2010; Sedzikowska and Szablewski, 2021
*TP53INP1*	Has a p53-independent antioxidant role; involved in mitochondrial function	Overexpression contributes to mitochondrial dysfunction and ROS generation, creating a cycle of oxidative stress	Mitochondrial dysfunction, oxidative stress	Autophagy, oxidative stress, apoptosis, cell cycle	Wang et al., 2017; Potenza et al., 2021; Kowalczyk et al., 2021; Aran and Singh, 2023
*HIGD1B*	Hypoxia-inducible gene highly expressed in pericytes	Promotes oxidative stress leading to ROS-mediated pericyte apoptosis, loss of BBB integrity, and microvascular dysfunction	Hypoxia, microvascular dysfunction	Mitochondrial metabolism	Winkler et al., 2022; He et al., 2020; Ding et al., 2020; Alkhalifa et al., 2023; Ogunshola and Tsao, 2023
*RFTN2*	Related to sphingolipids and lipid raft maintenance	Alters sphingolipid metabolism and lipid raft dynamics, potentially increasing Aβ formation through β- and γ-secretase localization	Lipid metabolism, Aβ formation	N/A	Giovagnoni et al., 2021; Quinville et al., 2021; Wang et al., 2024; Crews et al., 2009; Saeki, 2003; Simons and Ehehalt, 2002; Zhao et al., 2023
*ANLN*	Actin-binding protein involved in cytoskeletal regulation in oligodendrocytes	Disrupts actin cytoskeleton, impairing oligodendrocyte myelination, leading to loss of lipid-rich myelin and disrupted neuronal communication	Myelin formation, cytoskeleton regulation	Cell cycle, structural stabilization, immune response	Pandey et al., 2022; Maurin et al., 2018; Song et al., 2001; Iyer et al., 2024; Mot et al., 2018
*NEK7*	Interacts with NLRP3 inflammasome	Overexpression promotes chronic neuroinflammation via inflammasome activation, contributing to tau pathology and neuronal dysfunction	Neuroinflammation	Epigenetic, cell cycle, structural stabilization, metal binding and homeostasis	Xu et al., 2016; Swanson et al., 2019; Cheng et al., 2024; Zotova et al., 2010
*YES1*	SRC family tyrosine kinase linked to cell proliferation and possibly mTORC1/Syk pathways	Speculated to influence neuroinflammation through interactions with Syk and mTORC1, leading to impaired proteostasis and exacerbating AD pathology	Neuroinflammation, mTORC1/Syk pathways	Autophagy, immune response, vasculature, structural stabilization, lipid metabolism, synapse, epigenetic, proteostasis	Xu et al., 2018; Villait, 2020; Schweig et al., 2019; Perluigi et al., 2021
*TEAD2*	Part of Hippo pathway regulating neuronal development	May exacerbate tau-driven excitatory neuron loss in cortex and hippocampus, contributing to synaptic dysfunction and cognitive decline	Synaptic loss, tauopathy	Vasculature, epigenetic	Spires-Jones and Hyman, 2014; Mukhtar et al., 2020; Kumar et al., 2024; Fu et al., 2018; Chuang et al., 2014; Fu et al., 2017)
*DDIT4L*	Upregulated in stressed retinal ganglion cells	Contributes to axonal transport disruption via tau-related mechanisms	Axonal transport, tauopathy	N/A	Morquette et al., 2014; López-de-Eguileta et al., 2022; Kadavath et al., 2015; Berth and Lloyd, 2023
*DOCK1*	Implicated in neurite morphology in hippocampal neurons	Overexpression decreases neurite length, facilitating dystrophic neurites near Aβ plaques	Neurite integrity, Aβ plaque interaction	Autophagy, immune response, structural stabilization, apoptosis	Miyamoto and Yamauchi, 2009; Sanchez-Varo et al., 2011; Tsering and Prokop, 2023; Knowles et al., 1999; Sutherland et al., 2021

**Table 2:** Summary of top ten down-regulated genes from HDMA.

**Gene**	**Function/role**	**Identified relationship to Alzheimer's disease**	**Primary affected pathway**	**Biodomain annotations**	**References**
*C1QTNF4*	C1QNTF family member with implications in inflammation, glycolipid metabolism, vascular smooth muscle cells and interacts with nucleolin	Up-regulated intergenic transposable element (chr11: 47608036–47608220) suppresses expression of *C1QTNF4* in human iPSC neurons; potential causal relationship of AD-associated transposable element activation with neuroinflammation	Neuroinflammation, immune signaling	Apoptosis, immune response	Vester et al., 2021; Liu J. et al., 2023; Feng et al., 2024
*PCSK1N*	Encodes proSAAS protein which is distributed across the central nervous system and endocrine cells	proSAAS blocks fibrillation of β-amyloid and α-Syn, well-characterized neurodegenerative disease proteins	Amyloid aggregation inhibition, proteostasis	Proteostasis, synapse	Liu et al., 2012; Peinado et al., 2022
*VGF*	Contains variety of polypeptides with endocrine functions in physiological processes, homeostasis, and tumor pathogenesis	CSF levels of *VGF* decreases as AD progresses; *VGF* overexpression in the VGF/TLQP-62/BDNF/TrkB autoregulatory loop may slow or reverse neurodegeneration	Synaptic plasticity, neurotrophic signaling	Synapse, mitochondrial metabolism, proteostasis, immune response	Wang et al., 2022; Beckmann et al., 2020
*CTXN1*	Membrane protein enriched in rodent cerebral cortex, with predominant expression in cortical neurons	Identified as a predictive gene for AD and is notably highly dysregulated in the aging brain	Synaptic integrity	N/A	Coulter et al., 1993; Cheng et al., 2021
*LKAAEAR1*	Associated with synaptic plasticity	Current lack of established relationship with AD	N/A	N/A	Aczél et al., 2018
*TGFBR3L*	Encodes pituitary enriched membrane protein detected in gonadotroph cells, potentially involved in release of LH from gonadotroph pituitary neuroendocrine tumors	Dysregulation of HPG axis and elevated LH can alter structure of neuronal cells leading to promotion of neurodegeneration	Hormonal signaling, neuroendocrine regulation	Immune response	Kolnes et al., 2023; Meethal et al., 2005
*RPRML*	Intronless member of the Reprimo gene family expressed at low levels in tissues from GTEx database, may serve as biomarker for non-invasive detection of gastric cancer	Current lack of established relationship with AD	N/A	N/A	Alarcón et al., 2020
*SYBU-AS1*	RNA gene associated with lncRNA, regulates *SYBU* which produces protein Syntabulin notably involved in mitochondrial movement	Reduced mitochondrial trafficking to axonal terminals due to Syntabulin loss of function accelerates synaptic depression and slows the rate of synapse recovery	Mitochondrial transport, synaptic activity	N/A	Cai and Tammineni, 2016
*NRGN*	Encodes postsynaptic brain-specific protein, neurogranin, which regulates calmodulin-Ca^2+^ in neurons, associated with schizophrenia	Reduced synaptic neurogranin can lead to age-related cognitive decline through altered long-term potentiation, which is impaired in AD	Synaptic plasticity	N/A	Ruano et al., 2006; Saunders et al., 2023
*ZNF428*	Member of the zinc finger protein family	Current lack of established relationship with AD, various ZNFs are linked to AD through several pathways	N/A	Metal binding and homeostasis	Bu et al., 2021

### 4.1 Overexpressed genes implicate oxidative stress, microvascular abnormalities, lipid metabolism, and neuroinflammation as drivers of AD pathogenesis

#### 4.1.1 Oxidative stress promotes ROS leading to metabolic regulator deficiencies, mitochondrial dysfunction, and compromised microvascular formation

Oxidative stress has been implicated in AD as a pathophysiological mechanism where the imbalance between antioxidants and oxidants promotes increased free radicals (Huang et al., 2016). The accumulation of reactive oxygen species (ROS) can damage DNA leading to neuronal death (Narciso et al., 2016) and, ultimately, impair cognition processes in an aging brain (Ionescu-Tucker and Cotman, 2021). One of the top up-regulated genes was *FOXO4*, which is a member of the forkhead box class O (FOXO) family of transcription factors that are linked to oxidative stress (Storz, 2010; Santos et al., 2023). The FOXO transcription factors are involved in insulin signaling and cellular response to oxidative stress (Manolopoulos et al., 2010). In particular, *FOXO4* can bind to insulin-response elements and is involved in the regulation of insulin signaling pathways (Santos et al., 2023; Sedzikowska and Szablewski, 2021). One of the defining characteristics of sporadic AD is impaired brain insulin signaling (Erol, 2008), and insulin resistance in the brain microvasculature has been associated with beta-amyloid accumulation in mouse models of AD (Leclerc et al., 2022), suggesting that dysregulation of *FOXO4* may also contribute to cerebrovascular dysfunction in the disease. Concordant to insulin resistance, AD is marked by reduced cerebral glucose metabolism (Willette et al., 2015), and this disruption in metabolic homeostasis is further exacerbated by oxidative stress. Reactive oxygen species (ROS), a hallmark of AD pathology, can impair both insulin signaling and glucose uptake, initiating a self-reinforcing cycle of energy failure and neurodegeneration (Dhapola et al., 2024). Specifically, ROS-induced glucose hypometabolism impairs neuronal energy production and perpetuates further oxidative stress, ultimately compromising neuronal function and survival (Dhapola et al., 2024; Tramutola et al., 2016). Importantly, glucose hypometabolism has been identified as an early, even preclinical, signature of AD (Zilberter and Zilberter, 2017).

Oxidative stress also causes organelles to malfunction. *TP53INP1*, another top up-regulated gene, has been implicated in the shared pathologies between AD and type 2 diabetes through mitochondrial dysfunction and oxidative stress (Wang et al., 2017; Potenza et al., 2021). Mitochondrial dysfunction in the parahippocampal region of the brain has been associated with an early occurrence of AD (Wood, 2020). Mitochondrial dysfunction can arise from an excess of ROS, leading to mutations in mitochondrial DNA, interference with the mitochondrial respiratory chain, and alterations in mitochondrial defense mechanisms (Kowalczyk et al., 2021). As a result, compromised mitochondria may generate more ROS, creating a positive feedback loop that increases oxidative stress and further exacerbates mitochondrial dysfunction (Aran and Singh, 2023).

The dysfunction of organelles and cells caused by ROS and oxidative stress can also be initiated by hypoxia (D'Aiuto et al., 2022). Both hypoxia and oxidative stress are major risk factors that contribute to the pathogenesis of AD (Liu G. et al., 2023), while maintaining homeostasis in oxygen-dependent metabolism is key to regulating processes that exacerbate AD such as tau phosphorylation and Aβ aggregation (Zhang et al., 2018). Hypoxia-inducible factors (HIFs) can regulate hypoxia-inducible genes to adapt to the reduction in oxygen availability in order to preserve equilibrium (Chen et al., 2021). HIFs up-regulate genes to promote biological processes like angiogenesis (Lin et al., 2023). However, prolonged activation or overexpression of HIFs and hypoxia-responsive pathways in certain tissues may have maladaptive effects, but little is understood about this mechanism in the context of AD and neurodegeneration (Giordano, 2005; Chen et al., 2018). The top up-regulated gene in HDMA was *HIGD1B*, which is a hypoxia-inducible gene that is highly expressed in pericytes (Winkler et al., 2022). Pericytes are components of the neurovascular unit, responsible for regulating blood flow, blood-brain barrier protection, and angiogenesis (Wu et al., 2023; Ribatti et al., 2011). The overexpression of *HIGD1B* can promote oxidative stress, leading to a loss of pericytes predominantly from ROS-mediated pericyte apoptosis (He et al., 2020). The loss of pericytes in the white matter regions of the brain has been associated with various forms of dementia, including AD (Ding et al., 2020). Due to the loss of pericytes, the integrity of the blood-brain barrier becomes compromised which is a significant risk factor contributing to AD (Alkhalifa et al., 2023). In AD, microvascular dysfunction, such as pathological angiogenesis, can result from a loss of pericytes, leading to increased vessel permeability and decreased integrity, similar to the effects seen in tumor vascularization (Ogunshola and Tsao, 2023; Stapor et al., 2014).

Taken together, our findings are consistent with extensive reports that impaired insulin signaling and metabolic dysfunction are critical drivers of AD pathology. Our analyses further suggest that *FOXO4, TP53INP1*, and *HIGD1B* are key genes within these processes and that genetic variants influencing their expression contribute to AD pathology.

#### 4.1.2 High-ranking genes are implicated in lipid processes that contribute to Aβ formation and may compromise the production of essential lipid-rich myelin

Lipid homeostasis is an important process responsible for blood-brain barrier functionality, myelination, and neuronal receptor signaling (Chew et al., 2020). Dysfunction in lipid metabolism has been linked to AD pathology (Liu and Zhang, 2014) and may be one of the earliest neurobiological changes associated with AD and dementias (Katsel et al., 2007). A variety of lipid classes, including sphingomyelins, cholesterol esters, phosphatidylcholines, and triglycerides, are dysregulated in AD patients (Liu et al., 2021). The highly ranked up-regulated gene *RFTN2* has been identified as one of the top differentially expressed genes related to sphingolipids when comparing AD to control samples (Giovagnoni et al., 2021). Sphingolipids are responsible for the regulation of cell-to-cell interaction and cell death and can cause detrimental effects if disrupted (Quinville et al., 2021). The overexpression of sphingomyelinase, an enzyme that breaks down sphingomyelin, activates the ferroptosis signaling pathways, aggravating the symptoms of AD (Wang et al., 2024). Additionally, reducing the levels of sphingolipids can mitigate the accumulation of α-Synuclein, a naturally unfolded protein whose fragments can contribute to Aβ plaques, which can inhibit AD progression (Wang et al., 2024; Crews et al., 2009). Raftlins, the protein family containing *RFTN2*, may play a role in the formation and maintenance of lipid rafts, regions of the plasma membrane containing proteins and lipids capable of clustering (Saeki, 2003; Simons and Ehehalt, 2002). Lipid raft regions, which are abundant in cholesterol and sphingolipids, could act as anchors for β-secretase and γ-secretase, enzymes associated with Aβ formation (Zhao et al., 2023). Therefore, the up-regulation of *RFTN2* could contribute to Aβ accumulation in AD through its relationship to regulating sphingolipid functions and contributing to maintaining lipid rafts. Given the high ranking for *RFTN2* in HDMA, we suggest that reduced *RFTN2* expression may be associated with altered α-Synuclein processing and lipid raft dynamics, which in turn could play a role in modulating Aβ plaque formation.

In addition to the dysregulation of lipid-rich regions relevant to signaling pathways, the signaling pathways crucial for efficient neuronal communication can be disrupted through the lack of lipid-rich myelin. The up-regulated actin-binding protein gene *ANLN* is a cytoskeletal protein gene that is known to have elevated expression in oligodendrocytes in AD (Pandey et al., 2022). Oligodendrocytes are glial cells that are responsible for producing lipid-rich myelin to wrap around axons and insulate nerve cells in the brain and spinal cord (Simons and Nave, 2015; Bradl and Lassmann, 2009). In particular, it has been found that *ANLN* regulates the levels of GTPase RhoA in osteoclasts (Maurin et al., 2018). The Rho GTPases are molecular switches that play an important role in several signaling pathways such as regulating the actin cytoskeleton through the Rho proteins binding to an active GTP (Heasman and Ridley, 2008). It has also been shown using an inducible promoter construct that overexpression of RhoA promoted mitosis rather than entry into the G_2_/M phase, revealing that overexpressed RhoA disrupts the actin cytoskeleton with growth arrest, leading to disrupted actin dynamics that affect a cell's internal structure and shape (Song et al., 2001). Central to an oligodendrocyte's ability to produce myelin, the dynamics of the actin cytoskeleton powers necessary cell morphology to produce myelin (Iyer et al., 2024). It has been observed that the lack of insulation around axons of neurons contributes to AD pathology through dysfunctional synaptic communication. Indeed, oligodendrocyte and myelin disturbances have strong evidence in AD (Mot et al., 2018). Our HDMA results imply a putative causal role for *ANLN* on the dysfunction of oligodendrocytes, which we hypothesize disrupts the actin cytoskeleton and prevents oligodendrocytes from producing lipid-rich myelin in a manner dependent on RhoA. Thus, decreasing the expression of *ANLN* may act to mitigate AD pathology by supporting healthy myelin homeostasis.

#### 4.1.3 Genes implicated in neuroinflammation and chronic immune responses activate detrimental processes in the aging brain

Inflammation, particularly neuroinflammation, has been extensively implicated in AD (Akiyama et al., 2000). Chronic systemic inflammation has been linked to AD development, particularly affecting various AD pathologies including tau phosphorylation, blood brain barrier dynamics, and clearance of Aβ (Xie et al., 2022). In response to a wide range of AD pathologies, in particular Aβ accumulation, microglia produce proinflammatory cytokines which lead to a chronic state of neuroinflammation (Hansen et al., 2017). However, neuroinflammation is an exceedingly complex process that contributes to AD through diverse mechanisms, affecting and altering both cellular and molecular processes. *NEK7*, a top up-regulated gene, has been observed to directly interact with a domain of the NLRP3 inflammasome through potassium influx (Xu et al., 2016). The NLRP3 inflammasome is a protein complex that activates inflammatory responses such as caspase 1-dependent release of the proinflammatory cytokines IL-1β and IL-18 (Swanson et al., 2019). However, excessive activation of the NLRP3 inflammasome contributes to the progression of AD through its unregulated inflammatory response (Cheng et al., 2024). Decreasing the expression of *NEK7* has been shown to inhibit the chronic inflammatory response of the NLRP3 inflammasome, which we hypothesize could prevent general inflammation downstream effects such as disease-related tau proteins and neuronal dysfunction (Cheng et al., 2024; Zotova et al., 2010). Furthermore, silencing *NEK7* in microglia reduces inflammation in BV2 cells by inhibiting the activation of the TLR4/NF-kB pathway and NLRP3 inflammasome (Li et al., 2023). Moreover, cognitive impairment in APP/PS1 transgenic mice was alleviated through the silencing of *NEK7* (Li et al., 2023). These experiments demonstrate a critical putative causal role for *NEK7* in AD-related biology.

In addition to neuroinflammation-related processes, inflammation can occur through multiple pathways that target various tissues and organs and aggravate AD pathology. The high-ranking gene *YES1* is up regulated in the aged human frontal cortex and has been implicated as a molecular connection between aging and AD (Xu et al., 2018). *YES1* does not have a well annotated function in the brain but it is known to be involved in cell proliferation and cancer (Xu et al., 2018). In pancreatic ductal adenocarcinomas with oncogenic KRAS mutations, SRC family kinases *YES1* and *SRC* cooperate with spleen tyrosine kinase (Syk) to activate the mTORC1 pathway and may also function upstream to activate Syk in cell lines derived from genetically engineered mouse models (Villait, 2020). Syk itself is known to play a role in inflammation and immune responses through its upregulation in dystrophic neurites surrounding Aβ deposits, neurons affected by tau pathology, and activated microglia in individuals with AD (Schweig et al., 2019). It has been observed in a tauopathy mouse model that chronic SYK inhibition reduces tau buildup, neuroinflammation, and loss of neurons and synapses (Schweig et al., 2019). Along with SYK, the activation of mTORC1 in AD leads to the inhibition of protein quality control, preventing proteins from being folded to their native shape, resulting in decreased proteasome activity, a factor associated with aging and neurodegenerative disorders (Perluigi et al., 2021). Increased mTORC1 activation has been associated with brain insulin resistance and neuronal death in AD (Perluigi et al., 2021). Building on the interactions among *YES1*, Syk, and mTORC1 in pancreatic cancer cells, we propose that *YES1* plays a significant role in modulating neuroinflammation in neurodegenerative diseases. Additionally, the YAP1, *YES1*-associated transcriptional regulator, ranks relatively highly among top up-regulated mediators.

#### 4.1.4 The complex interactions among Aβ plaque accumulation, tauopathies, and the functions of high-ranking genes lead to neuronal death and loss of synaptic communication

*TEAD2* is a highly ranked up-regulated gene involved in pathways associated with synaptic loss and neuronal death. Neuronal and synaptic dysfunction with Aβ accumulation is associated with an increase in calcium concentration, caspase-3 activation, and downstream internalization of synaptic receptors (Spires-Jones and Hyman, 2014). Although tau's association with synaptic dysfunction is not as well studied, evidence from mitochondrial dynamics, microtubule-based transport, and tau's involvement downstream of Aβ implicates a role in synaptic dysfunction (Spires-Jones and Hyman, 2014). In neuronal stem cells, the role of Yap1/Taz coactivators and Teads within the Hippo pathway was explored in the context of cortical development (Mukhtar et al., 2020). The expression of *TEAD2* blocks Tbr1^+^ neuron production (Mukhtar et al., 2020). The progressive loss of neurons in a specific brain region is an early manifestation of AD, where loss typically first occurs in the entorhinal cortex within the hippocampal formation and progresses to other regions of the brain leading to neuronal death (Kumar et al., 2024). Additionally, pathological tau accumulation occurs in excitatory neurons, which up-regulate *Tbr1*, not only in the entorhinal cortex but in regions affected as AD progresses (Fu et al., 2018; Chuang et al., 2014). The accumulation of tau proteins forms tangles which disrupts the balance between inhibitory and excitatory neurons that contributes to death of synaptic connections (Fu et al., 2017). We therefore speculate that variants affecting *TEAD2* expression along with tau accumulation can exacerbate the loss of excitatory neurons in various brain regions crucial for memory and cognitive functions.

The gene *DDIT4L* (*REDD2*) is an up-regulated gene from HDMA that is also upregulated in injured retinal ganglion dendrites (Morquette et al., 2014). Retinal ganglion cells are particularly vulnerable to neurodegenerative diseases in humans and animal models due to an early manifestation of AD appearing as neuronal impairment (Agostinone and Di Polo, 2015). In particular, it has been demonstrated that damage sustained to the retinal ganglion cell layer leads to increased cerebrospinal fluid phosphorylated-181-Tau and total Tau (López-de-Eguileta et al., 2022). The physiological role for tau is the stabilization of neuronal microtubules, and neurofibrillary tangles, which are aggregates of hyperphosphorylated tau proteins, destabilize microtubules and cause the perturbation of axonal transport (Kadavath et al., 2015). Indeed, the result of axonal swellings and dystrophic axons is a prominent feature of the disruption of axonal transport, which was found to be an early event in postmortem brains of AD mice (Berth and Lloyd, 2023). Since injury in retinal ganglion cells has been shown to upregulate *DDIT4L*, and such damage can promote alterations in tau protein, which in turn may destabilize microtubules and perturb axonal transport. Together, these processes could establish a feedback cycle, where persistent axonal injury sustains *DDIT4L* upregulation as part of the cellular attempt to mitigate damage.

The gene *DOCK180* (*DOCK1*) is a top up-regulated gene from HDMA whose overexpression is implicated in decreasing the length of neurites in hippocampal neurons (Miyamoto and Yamauchi, 2009). Dystrophic neurites associated with Aβ plaques occur prior to neuronal death and are an early manifestation of AD (Sanchez-Varo et al., 2011). Aβ plaques are thought to be potentially responsible for the formation of dystrophic neurites (Tsering and Prokop, 2023). For example, neurites that pass-through accumulations of Aβ plaques can disrupt synchronous activation of neural systems by losing their straight shape geometry (Knowles et al., 1999). In neurodegenerative diseases, older neurons have reduced neurite regenerative capacity and genetic expression of genes like *DOCK1* may contribute to a decrease in neurite length correlated with Aβ plaques and exacerbating cognitive decline (Sutherland et al., 2021).

Notably, while *TEAD2, DDIT4L*, and *DOCK1* are involved in complex interactions involving Aβ plaque accumulation and tauopathies leading to synaptic dysfunction, their upregulation within this domain contrasts with the broader downregulation of genes involved in neurotransmission and synaptic maintenance. This difference may indicate participation in stress-response or remodeling pathways engaged during neurodegeneration, rather than in maintaining normal synaptic function.

### 4.2 Perturbagens ranging from genes to small-molecule compounds implicate new strategies to counter AD pathologies

The ultimate value of causal hypothesis generation is in the prioritization of novel hypotheses to control disease processes. Among the top perturbagens were multiple promising candidates (Table 3), including six perturbagens with direct evidence for efficacy in AD models (green rows), seven perturbagens with evidence for efficacy in an AD-related pathway (yellow rows), and five perturbagens with limited direct evidence but significant score in Clue.io. These results demonstrate that the ConnectivityMap approach applied to HDMA-derived signatures can be used to prioritize promising therapeutic leads for highly polygenic disorders.

**Table 3:** Top perturbagens from Clue.io.

**Clue.io perturbagen**	**Perturbagen type**	**Known biological pathway(s)**	**Relation to neurodegenerative diseases**	**Hypothesized pathway to counter AD**	**References**
*ZNF384*	Silenced gene expression (shRNA for LoF of gene)	Regulates transcription factors including PPARG, ZNF415, HLX, ANHX	*ZNF384* is involved in inflammation and metabolism processes, a pathogenesis prevalent in AD	Downregulation of *ZNF384* may suppress neural inflammation	Liu et al., 2022
*GPR141*	Silenced gene expression (shRNA for LoF of gene)	Regulates cell proliferation through p-mTOR/p53 axis	*GPR141* is upregulated in AD microglia compared to control microglia	Downregulation of *GPR141* may promote greater microglia homeostasis to reduce neuroinflammation	Parija et al., 2023; Srinivasan et al., 2020; Miao et al., 2023
*SMAD3*	Silenced gene expression (shRNA for LoF of gene)	Transforming growth factor-beta signaling pathway	Inhibition of *SMAD3* in an APP/PS1 mouse model enhances Aβ clearance by peripheral macrophages	Blocking *SMAD3* expression may increase Aβ plaque clearance in humans	Miyazono, 2000; Xu et al., 2021
*TYK2*	Silenced gene expression (shRNA for LoF of gene)	Promotes components of type I and type III interferon signal pathways	Tyrosine phosphorylation of STAT3 is elevated in the cortex and hippocampus of APP/PS1 transgenic mice; *TYK2*-STAT3 mediates Aβ-dependent neuronal death in AD models	Downregulation of *TYK2* may prevention of STAT3 activation and Aβ-induced cell death	Leitner et al., 2015; Wan et al., 2010
AZD-5438	Compound	Potent CDK 1, 5, 9 inhibitor	AZD-5438 prevents neuronal death and mitochondrial dysfunction in human iPSC-derived cortical and midbrain neurons	AZD-5438 treatment can increase inhibition of GSK-3α/β and CDKs which may be neuroprotective	Byth et al., 2009; Shi et al., 2023
*ABCB1*	Silenced gene expression (shRNA for LoF of gene)	MDR1-TypeP-Glycoproteins protect the brain against accumulation of toxic xenobiotics	*ABCB1* codes for a blood-brain barrier efflux transporter P-glycoprotein that pumps out Aβ	Up-regulation of *ABCB1* may enhance Aβ clearance to reduce Aβ deposition	Müller et al., 2003; Kohen et al., 2011; ElAli and Rivest, 2013
BRD-A01593789	Compound	Chlormadinone acetate suppresses gonadotropin secretion, 5 alpha-reductase inhibitor via blocking androgen receptors	5 alpha-reductase inhibitor-induced dihydrotestosterone deficiency can increase the risk of dementia in elderly men, low androgen levels may cause cognitive impairment	5 alpha-reduced androgens may be neuroprotective against AD	Raudrant and Rabe, 2003; Xu et al., 2023; Cai and Li, 2020; Mendell et al., 2020
*DNAJC5B*	CRISPR for LoF	Regulates proper folding of other proteins along with heat shock protein 70	*DNAJC5* encodes CSPα	CSPα is reduced in the forebrain in the initial stages of synaptic degeneration; blocking *DNAJC5* may be neuroprotective in the forebrain	Suh et al., 1998; Tiwari et al., 2015
*TM7SF2*	Silenced gene expression (shRNA for LoF of gene)	Endoplasmic reticulum protein responsible for reducing C14 unsaturated sterol intermediates during cholesterol synthesis	*TM7SF2* controls an anti-inflammatory loop (NF-κB activation and TNFα upregulation) which may contribute to neurodegenerative diseases	Downregulation of *TM7SF2* may decrease pro-inflammatory cytokines which may neuroinflammation	(Bartoli et al., 2016; Bellezza et al., 2013; Tilstra et al., 2011)
*HSF2*	Over-expression of wild-type gene	Activated when the ubiquitin-proteasome system is inhibited, binds heat-shock element promoters, driving expression of heat-shock genes	*HSF2* accelerates the accumulation of ubiquitylated misfolded proteins when deleted in mouse model of polyQ disease	Up-regulation of *HSF2* may promote cell survival under proteotoxic stress and maintain cell-cell adhesion, and decrease accumulation of Aβ and tau proteins	Mathew et al., 1998; Neef et al., 2011; Joutsen et al., 2020
*ELF1*	Over-expression of wild-type gene	Regulates immune responses including expression of T-cell-specific genes	Up-regulated expression of *ELF1* in glioma tissues promotes tumor progression by regulating the MEIS1/GFI1/FBW7 axis	Up-regulation of *ELF1* may modulate neuroinflammatory pathways in AD	Rellahan et al., 1998; Cheng et al., 2020
*PSMD10*	Silenced gene expression (shRNA for LoF of gene)	Directly binds to CDK4, enhancing kinase activity by displacing CDK inhibitors	*PSMD10* involved in neurogenesis of human neural progenitor cells by stabilizing β-Catenin	*PSMD10* may promote the expression of autophagy gene, *ATG7*, and suppress Aβ accumulation	Nagao et al., 2003; Sahu et al., 2019; Liu et al., 2024; Nilsson et al., 2014
RITA	Compound	Anti-tumor small molecule agent that targets the p53 pathway by disrupting the p53-MDM2 interaction	Specific inhibition of p53-MDM2 interaction with a small molecule for cancer rescues both the neurogenic and cognitive deficits of fragile X syndrome mice	The MDM2-inhibitor nutlin-3 blocked APP overexpression-induced spine loss and preserved normal spine density rat hippocampal neurons	Issaeva et al., 2004; Li et al., 2016; Martinez et al., 2024
XMD-132	Compound	–	–	Further research needed	
BRD-K80648041	Compound	–	–	Further research needed	
BRD-K06652922	Compound	–	–	Further research needed	
BRD-K69886508	Compound	–	–	Further research needed	
*PI4K2B*	Silenced gene expression (shRNA for LoF of gene)	Member of the type II PI4 kinase protein family whose protein is involved in early T cell activation	–	Further research needed	Sinha et al., 2013
*GMDS*	Silenced gene expression (shRNA for LoF of gene)	Mutations in gene result in resistance to TRAIL-induced apoptosis	–	Further research needed	Moriwaki et al., 2009

The perturbagens are divided among their extensive evidence in AD and related neurodegenerative diseases (green), moderate evidence in association with AD and neurodegenerative diseases (yellow), and lack of evidence with AD and neurodegenerative diseases (red).

Among the gene-targeted perturbations with substantial evidence supporting their relevance to AD pathophysiology, *SMAD3* and *HSF2* are of particular interest. Targeted knockdown of *SMAD3*, a key mediator of transforming growth factor-beta signaling, has been demonstrated to enhance Aβ clearance via peripheral macrophages in APP/PS1 mouse models, leading to a reduction in neuroinflammation and amelioration of cognitive deficits (Miyazono, 2000; Xu et al., 2021). Likewise, overexpression of *HSF2*, a component of the heat shock response, has been shown to mitigate proteotoxic stress by preserving cell-cell adhesion and limiting the accumulation of misfolded proteins, including Aβ and tau, both of which are central to AD pathology (Mathew et al., 1998; Neef et al., 2011; Joutsen et al., 2020) and represented in our composite phenome score. In contrast, the gene-targeted perturbation of *ELF1* is supported by moderate evidence. *ELF1* upregulation has been implicated in the regulation of neuroinflammatory pathways through the MEIS1/GFI1/FBW7 axis in glioma models, but its direct role in AD remains to be elucidated (Rellahan et al., 1998; Cheng et al., 2020).

On the other hand, compounds such as XMD-132 currently lack any reported association with AD, and their mechanistic relevance to neurodegenerative disease processes has not yet been established. Additionally, three of the BRD compounds do not have any notable relation to AD or neurodegenerative diseases which is an area to be extensively researched. Of particular note is the small-molecule compound RITA, originally developed as an anti-tumor small molecule agent targeting the p53-MDM2 interaction. Emerging evidence suggests that MDM2 inhibition may have therapeutic potential in AD-related contexts, as RITA has been shown to rescue both neurogenic and cognitive deficits in a fragile X syndrome model and to prevent dendritic spine loss induced by APP overexpression in hippocampal neurons (Issaeva et al., 2004; Li et al., 2016; Martinez et al., 2024). These findings highlight the potential of anti-cancer compounds for repurposing in the treatment of neurodegenerative diseases such as AD.

### 4.3 Limitations and caveats of the current study and future direction

We note several caveats to the current study are important when considering our results. First, in pre-processing the transcriptomic data, we opted not to control for purely technical factors for two reasons. HDMA provides significant constraint on the learned transcriptomic signature, namely that it has to be simultaneously correlated with kinship (i.e., heritable) and correlated with phenotypic outcomes. *A priori* we do not expect technical factors to be correlated in this way. However, to the extent they are, the study is confounded and we cannot distinguish between technical and real associations. Such confounding is not an idle concern, as Tian et al. (2024) have shown that RNA Integrity Number (RIN), a putatively purely technical measure of sample quality, is in fact correlated with AD pathology, particularly in the ROSMAP dataset. The downstream characterization of this signature is intended to bolster a causal, as opposed to the confounded, interpretation, but more work, including analyses of additional cohorts and mechanistic studies, remains. A second caveat is that from a genetic perspective, by starting with the kinship matrix, our analysis is limited by the fact that it does not implicate individual variants and cannot distinguish between cis- and trans-effects of variants on gene expression. We speculate that the transcriptomic signature we observe is driven dominantly by trans-effects, based on the lack of enrichment of putative eQTL target genes (Supplementary Figure 4) and the highly polygenic architecture of LOAD, but verifying this is beyond the scope of current data. A final caveat is that cross-sectional studies such as ROSMAP can only support inference of putative causal processes based on patterns of correlations that are consistent with causation. However, validating causation requires intervention studies.

Despite the limitations above, this study proves concept that putative mediators of genetic risk for AD can be inferred from high-dimensional omics profiles. Our findings are well supported by existing mechanistic studies in the literature and imply novel treatment approaches. Further studies are required to elaborate specific mechanisms implied by our analysis. Specifically, currently genomics, transcriptomics, and phenotypes were provided to HDMA to capture mediators, however integrating omics layers beyond these such as metabolomics, proteomics, and epigenetics may reveal the identification of mediators contributing to the pathogenesis of AD (Pérez-González et al., 2024). At the system level using genomics, transcriptomics, and phenotypes, complex feedback loops exist among metabolic stress, neuroinflammation, hypoxia, angiogenesis, and neuron death, which act at the level of tissues and circuits. Using HDMA, we have prioritized high-quality candidate genes and compounds as potential therapeutic leads.

## Data Availability

Publicly available datasets were analyzed in this study. This data can be found here: the datasets analyzed for this study can be found in Synapse as syn3157325, syn23650893, syn3191087, and syn8691134 under AD Knowledge Portal (https://adknowledgeportal.org).

## Supplementary material

Supplementary Table 1 GSEA results for AD biodomains using HDMA-derived transcriptomic rankings.

Supplementary Table 2 HDMA ranked genes featuring up-regulated genes (positive scores) and down-regulated genes (negative scores) with respective gene name and Ensembl ID.

Supplementary Figure 1 PCA analysis of ROSMAP phenome. **(A)** Cumulative variance explained across 20-dimensional phenome shows that the first two principal components explain 48% of the variance across all measures. **(B)** PC1 is defined by the contrast between MMSE and CERAD scores (lower in AD) and the remaining measures (higher in AD). **(C)** PC2 is defined mostly by the contrast between Braak scores and the remaining measures.

Supplementary Figure 2 Network structure of positive loading genes in specific GO terms and corresponding AD biodomains. Leading edge genes from gene-set enrichment analysis of transcript loadings sorted in descending order of GO terms from the AD sub-biodomains immune response (IR), structural stabilization (SS), and vasculature (Va).

Supplementary Figure 3 Network structure of negative loading genes in specific GO terms and corresponding AD biodomains. Leading edge genes from gene-set enrichment analysis of transcript loadings sorted in ascending order of GO terms from the synapse (Sy) AD sub-biodomain.

Supplementary Figure 4 Analysis of loadings for genes detected through genome-wide association studies (GWAS) and expression quantitative trait locus colocalization analysis. The (absolute) HDMA-derived loadings of genes prioritized by Bellenguez et al. using GWAS colocalization analysis are not significantly higher than random genes from the distribution of loadings, either for the genes known to be involved prior to their study or the novel ones implicated by their analysis.
