# Supplementary figures and images for: Identifying transcriptomic signatures that mediate the causal effect of genotype on Alzheimer's disease
Source: Front Neurosci. 2026 Mar 11;20:1716828. doi: 10.3389/fnins.2026.1716828 (PMC13044590; doi:10.3389/fnins.2026.1716828)

A

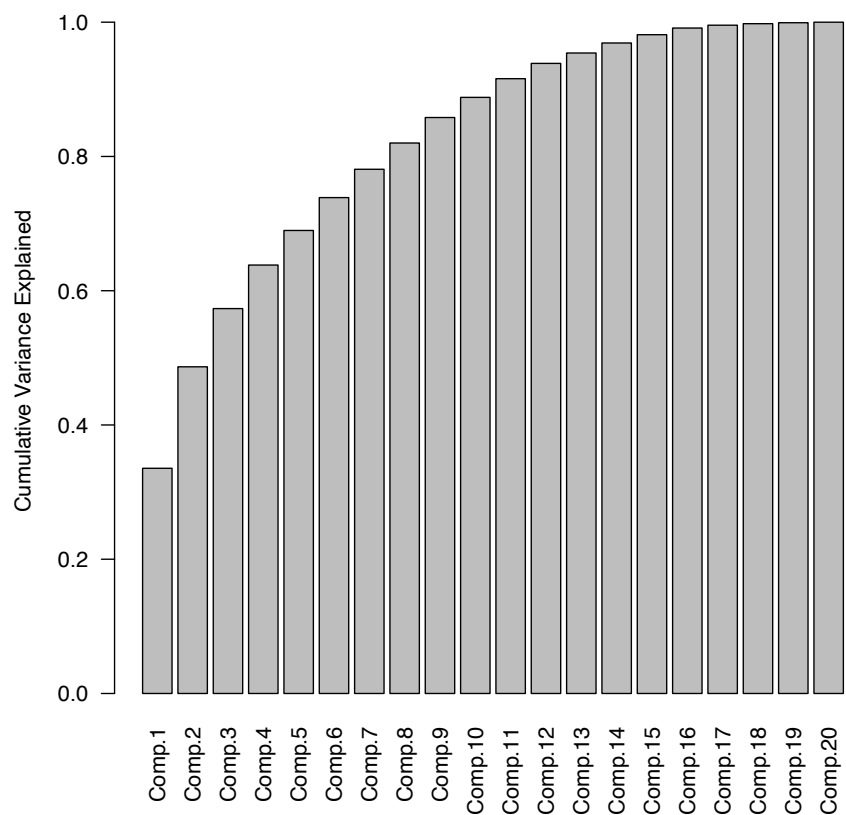

B

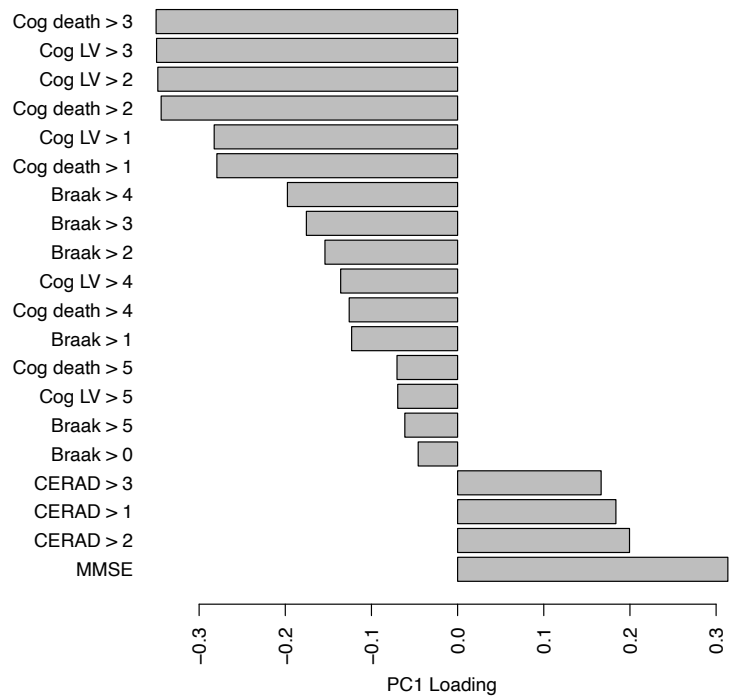

C

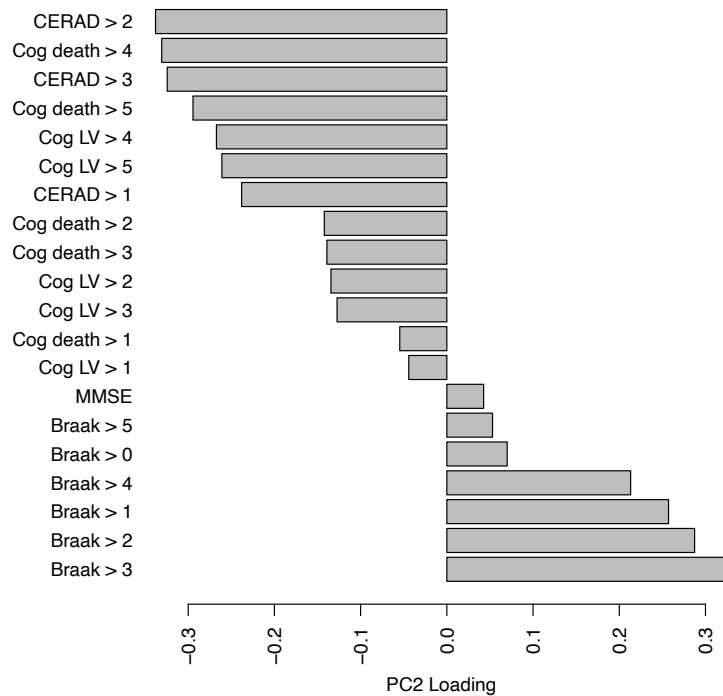

Supplement: Supplementary Figure 1 — PCA analysis of ROSMAP phenome. (A) Cumulative variance explained across 20-dimensional phenome shows that the first two principal components explain 48% of the variance across all measures. (B) PC1 is defined by the contrast between MMSE and CERAD scores (lower in AD) and the remaining measures (higher in AD). (C) PC2 is defined mostly by the contrast between Braak scores and the remaining measures. [file Image_1.pdf]

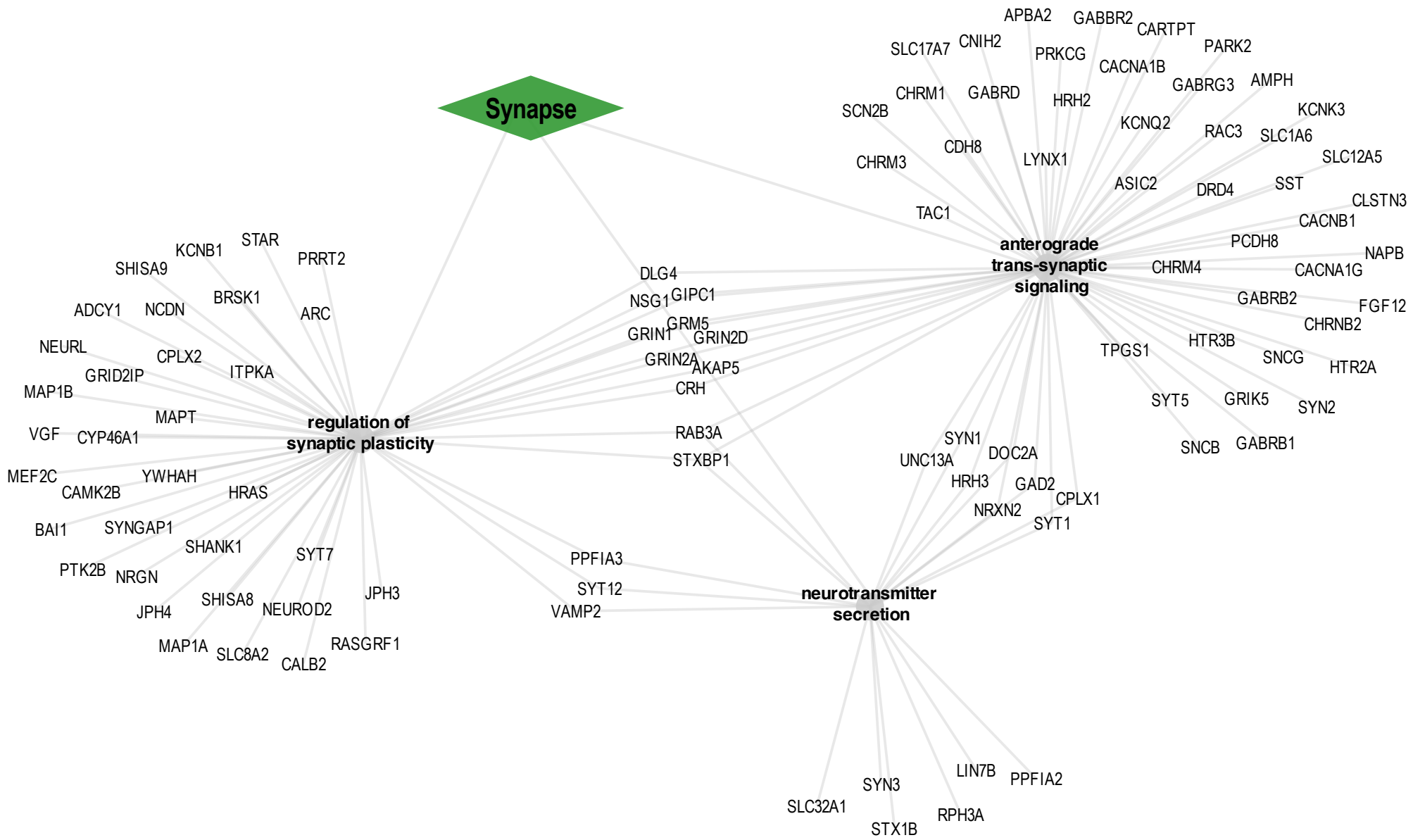

Supplement: Supplementary Figure 3 — Network structure of negative loading genes in specific GO terms and corresponding AD biodomains. Leading edge genes from gene-set enrichment analysis of transcript loadings sorted in ascending order of GO terms from the synapse (Sy) AD sub-biodomain. [file Image_3.pdf]

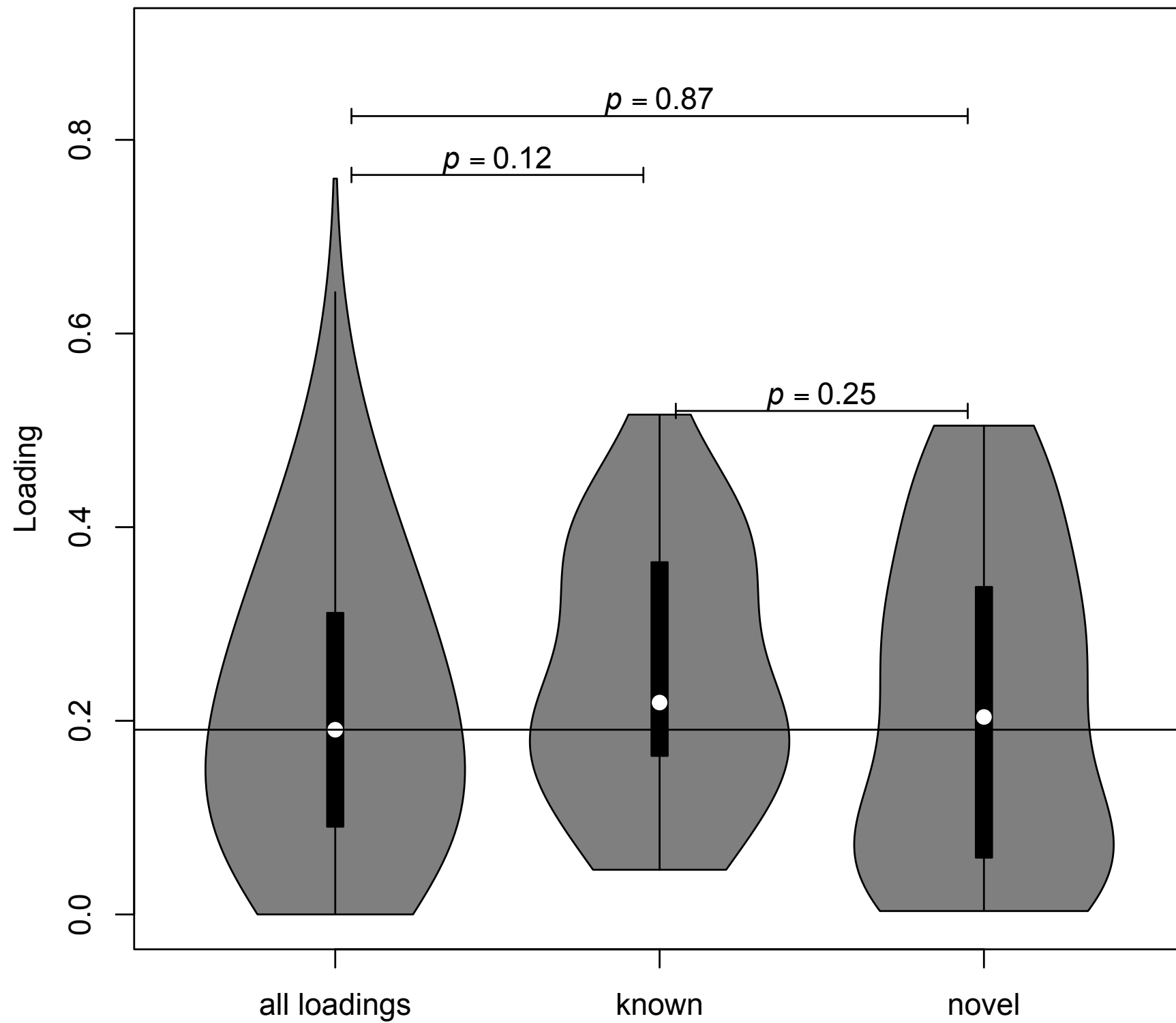

Supplement: Supplementary Figure 4 — Analysis of loadings for genes detected through genome-wide association studies (GWAS) and expression quantitative trait locus colocalization analysis. The (absolute) HDMA-derived loadings of genes prioritized by Bellenguez et al. using GWAS colocalization analysis are not significantly higher than random genes from the distribution of loadings, either for the genes known to be involved prior to their study or the novel ones implicated by their analysis. [file Image_4.pdf]
