# Supplementary material for: Identifying transcriptomic signatures that mediate the causal effect of genotype on Alzheimer's disease
Source: Front Neurosci. 2026 Mar 11;20:1716828. doi: 10.3389/fnins.2026.1716828 (PMC13044590; doi:10.3389/fnins.2026.1716828)
Supplement: Supplementary Figure 2 — Network structure of positive loading genes in specific GO terms and corresponding AD biodomains. Leading edge genes from gene-set enrichment analysis of transcript loadings sorted in descending order of GO terms from the AD sub-biodomains immune response (IR), structural stabilization (SS), and vasculature (Va). [file Image_2.pdf]

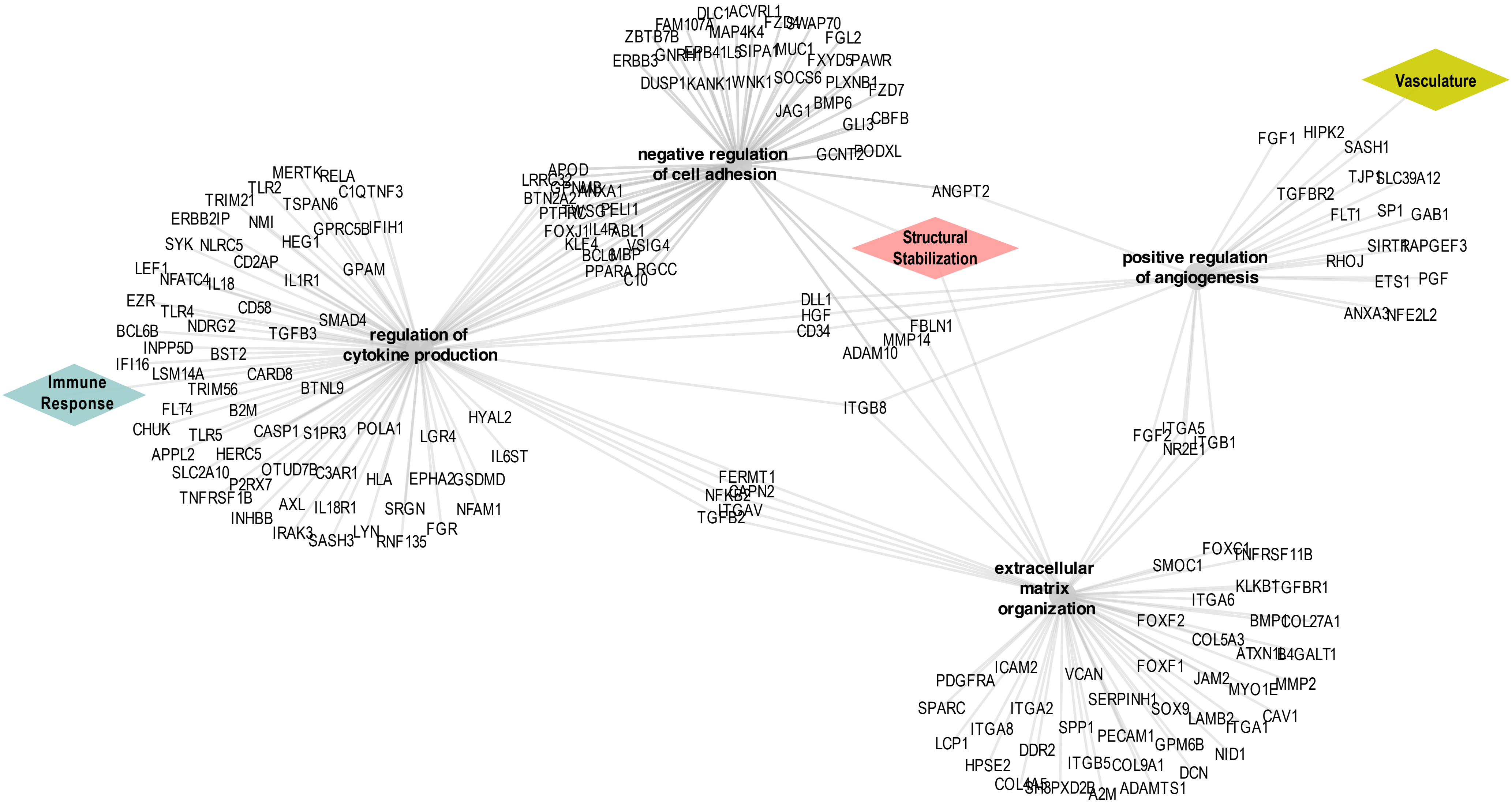

Immune Response
Vasculature
Structural Stabilization
regulation of cytokine production
negative regulation of cell adhesion
positive regulation of angiogenesis
extracellular matrix organization
DLC1
ACVRL1
FAM107A
SWAP70
MAP4K4
ZBTB7B
FGL2
ERBB3
EPB41L5
SIPA1
MUC1
FXYD5
PAWR
DUSP1
KANK1
WNK1
SOCS6
PLXNB1
FZD7
JAG1
BMP6
GLI3
CBFB
PODXL
APOD
LRRC32
ANXA1
BTN2A2
PELI1
FOXJ1
ABL1
KLF4
VSIG4
MBP
PPARA
RGCC
C10
ANGPT2
FGF1
HIPK2
SASH1
TJP1
SLC39A12
TGFBR2
FLT1
SP1
GAB1
RAPGEF3
RHOJ
ETS1
PGF
ANXA3
NFE2L2
DLL1
HGF
CD34
FBLN1
MMP14
ADAM10
ITGB8
FGF2
ITGA5
NR2E1
ITGB1
MERTK
RELA
TLR2
C1QTNF3
TRIM21
TSPAN6
ERBB2IP
NMI
GPRC5B
IFIH1
SYK
NLRC5
HEG1
LEF1
CD2AP
GPAM
NFATC4
IL18
IL1R1
EZR
TLR4
CD58
SMAD4
BCL6B
NDRG2
TGFB3
INPP5D
BST2
IFI16
LSM14A
CARD8
TRIM56
BTNL9
FLT4
B2M
HYAL2
CHUK
TLR5
CASP1
S1PR3
POLA1
LGR4
APPL2
HERC5
IL6ST
SLC2A10
OTUD7B
C3AR1
P2RX7
HLA
EPHA2
GSDMD
TNFRSF1B
AXL
IL18R1
SRGN
NFAM1
INHBB
IRAK3
LYN
FGR
SASH3
RNF135
FERMT1
NFKB2
CAPN2
ITGAV
TGFB2
FOXC1
SMOC1
TNFRSF11B
KLKB1
TGFBR1
ITGA6
BMP1
COL27A1
FOXF2
COL5A3
ATXN1L
B4GALT1
ICAM2
VCAN
FOXF1
JAM2
MYO1E
MMP2
PDGFRA
SERPINH1
SOX9
LAMB2
CAV1
SPARC
ITGA2
ITGA1
ITGA8
SPP1
PECAM1
GPM6B
LCP1
DDR2
NID1
HPSE2
ITGB5
COL9A1
DCN
SH3PXD2B
A2M
ADAMTS1
